# Supplementary material for: Guidance to best tools and practices for systematic reviews
Source: Syst Rev. 2023 Jun 8;12:96. doi: 10.1186/s13643-023-02255-9 (PMC10248995; doi:10.1186/s13643-023-02255-9)
Supplement: Supplementary file 1 — Additional file 2A. Overviews, scoping reviews, rapid reviews and living reviews. [file 13643_2023_2255_MOESM1_ESM.pdf]

Additional File 2A: Overviews, scoping reviews, rapid reviews, and living reviews

Overviews or umbrella reviews

These are syntheses of data reported by systematic reviews. Their increasing popularity in the medical literature and their use in clinical practice guidelines reflects the current glut of intervention systematic reviews. They are classified as "overviews" by Cochrane[1] and "umbrella reviews" by JBI[2] but are also referred to as "reviews of reviews" and "meta-reviews." We use the terms overview and umbrella review interchangeably. The two most common applications of umbrella reviews are: 1) summaries of treatment effects of multiple interventions for one or more diseases of interest; and 2) epidemiological associations of exposures.[3] They can also summarize secondary evidence that reports on prevalence[4] and evaluations of clinical tools for their diagnostic accuracy.[5]

Authors considering developing an overview or umbrella review must first determine if undertaking a new systematic review would be more appropriate.[1,3] Undertaking a new systematic review instead of an overview is recommended when: 1) when existing reviews do not address the research question adequately; and 2) significant numbers of existing reviews are outdated or methodologically flawed. If the authors' purpose is to answer a different question from those posed in the included systematic reviews (eg, outcomes relating to a subpopulation, or subsets of interventions or outcomes), it is appropriate for authors of overviews to re-extract and re-analyze outcome data (eg, using meta-analysis) from a set of non-overlapping systematic reviews.[1]

If their question is the same or similar enough to the one posed in the included systematic reviews, overview authors must consider two levels of data: the data presented in the included systematic reviews, and the data reported in the primary studies synthesized by these systematic reviews. This requires reporting elements and methods not applicable to conventional systematic reviews.[4,5] For example, methods are required to deal with overlapping and discordant outcome data reported by the included systematic reviews.[6-8] In addition, risk of bias assessments of primary studies may be missing, inadequately reported, or reported differently across the included systematic reviews. Another methodological challenge is encountered when overview authors include supplemental evidence from primary studies; there is ongoing debate about whether, when, and how this should be done.[7,9] This specific issue is addressed in more detail in Part 2 of the main text.

Not surprisingly, concerns about the rigor of umbrella reviews have been raised.[10-13] These prompted development of a reporting guideline.[7,14] While methodological guidance for overviews has been accumulating, broad consensus on methods has not yet established. As such, it is imperative that authors of umbrella reviews develop *a priori* decision rules in a detailed protocol.[9] At this time, methods for overviews from Cochrane[1] are limited to research questions about intervention effects. The JBI[2] methodology can theoretically be applied to any systematic review type but the types to be included in an umbrella review must be stipulated clearly in an a priori protocol. Additional sources of guidance are available for umbrella reviews pertaining to non-intervention topics.[3]

Scoping reviews

Scoping reviews generally have broad questions and may investigate complicated concepts. They are particularly applicable to emerging evidence on a topic and to a body of evidence that is of a complex or heterogeneous nature not amenable to a more precisely circumscribed systematic review of the evidence. Guidance is available for authors who are unsure if they should undertake a scoping review or a systematic review.[15] JBI also offers a YouTube video to assist in making this determination.[16] Appropriate reasons for undertaking a scoping review are listed on Table AF2A. Of note, scoping reviews can offer practical benefits to potential systematic review authors. They can assist in the development and confirmation of a priori inclusion criteria and ensure that the research questions of a systematic review can be answered by the available, relevant evidence.[15]

Table AF2A: Possible purposes of a scoping review[a]

To identify the types of available evidence in a given field.
To identify and analyze knowledge gaps.
To clarify key concepts/definitions in the literature.
To examine how research is conducted on a certain topic or field.
To identify key characteristics or factors related to a concept.
To inform the development of a systematic review.

[a]Adapted from Munn and colleagues[15]

Tricco and colleagues investigated the conduct and reporting of scoping reviews,[17] which led to the publication of a reporting guideline (PRISMA-ScR).[18] JBI provides methodological guidance for scoping reviews.[19] Scoping reviews include many of the same components as systematic reviews but apply different methods. For example, the recommended format for a scoping review research

question describes the "Population," "Concept," and "Context."[19] Results in a scoping review may be presented as a "map" of the data using a logical diagrammatic or tabular formats; these show at-a-glance the quantity and types of research available on a particular topic.[20] Scoping reviews provide a wide view of the literature and identify gaps. They typically do not address risk of bias concerns or include specific evaluations of other factors considered in judgments about the overall certainty of a body of evidence for specific outcomes.

Rapid reviews

Rapid reviews are not new but are now considered a viable part of the review literature. When a topic is particularly time-sensitive, a rapid review may be considered. Cochrane and the World Health Organization (WHO) have developed similar methodologies for this type of review.[21-23] In general, each review component has been streamlined without losing overall integrity. Limiting the scope of the research question (while still including stakeholders) and only conducting a meta-analysis when absolutely necessary are two key components. Tricco and colleagues emphasize the need for absolutely transparent methods as these methods have not been evaluated.[22] They also offer suggestions for technology that can assist in the production of a rapid review. Dobbins offers guidance on equity and qualitative questions.[23]

Living reviews

This is a methodology for updating evidence syntheses applicable to any type of review. Living reviews are most appropriate when the topic is essential for decision-making, the current evidence is of low or very low certainty, new information may clarify or change the findings, and there is anticipation that such new information may keep accruing in the near future.[24] A living review is updated when relevant new evidence is published; thus, knowledge of registered, ongoing studies can inform expectations about new data availability. This may be as often as monthly or typically longer (eg, 6-month) intervals. A living review is often connected with living guidelines[24] and online publication may help with their prompt dissemination.[25]

The standard approach to living reviews involves frequent updates to intervention systematic reviews, which traditionally rely upon results of meta-analyses (ie, pairwise treatment comparisons) to inform their conclusions.[26] The utility of such a narrow evidence focus in living reviews has been questioned.[27] In addition, concerns have been raised about their long-term feasibility given continuous evidence updating requires sophisticated technological resources and long-term commitments from author groups.[28] These and other limitations of living reviews, some of which

also apply to conventional systematic reviews of interventions, have inspired innovative approaches such as the Live Cumulative Network Meta-analysis[29] and Living Interactive Evidence Synthesis[28] frameworks. More information about the network meta-analytical approach to quantitative syntheses is found in Part 4 of the main text.

REFERENCES

1. Pollock M, Fernandez R, Becker LA, Pieper D, Hartling L. Chapter V: Overviews of reviews. In J. Higgins, J. Thomas, J. Chandler, M. Cumpston, T. Li, M. Page, et al. (Eds.), *Cochrane handbook for systematic reviews of interventions* [internet]. Cochrane; 2022 [cited 2022 Mar 7]. Available from: https://training.cochrane.org/handbook/current/chapter-v.
2. Aromataris E, Fernandez R, Godfrey C, Holly C, Khalil H, Tungpunkom P. Chapter 10: umbrella reviews. In: Aromataris E, Munn Z, editors. *JBI Manual for Evidence Synthesis* [internet]. JBI; 2020 [cited 2022 Jul 11]. Available from: https://synthesismanual.jbi.global.
3. Belbasis L, Bellou V, Ioannidis JPA. Conducting umbrella reviews. BMJ Med. 2022;1(1):e000071.
4. Choi J, Price J, Ryder S, Sikand D, Solmi M, Kisely S. Prevalence of dental disorders among people with mental illness: an umbrella review. Aust N Z J Psychiatry 2022;56:949–63.
5. Sambrook Smith M, Cairns L, Pullen LSW, Opondo C, Fellmeth G, Alderdice F, et al. Validated tools to identify common mental disorders in the perinatal period: a systematic review of systematic reviews. J Affect Disord. 2022;298(Pt A):634-43.
6. Lunny C, Brennan SE, McDonald S, McKenzie JE. Toward a comprehensive evidence map of overview of systematic review methods: paper 1-purpose, eligibility, search and data extraction. Syst Rev. 2017;6(1):231.
7. Pollock M, Fernandes RM, Pieper D, Tricco AC, Gates M, Gates A, et al. Preferred Reporting Items for Overviews of Reviews (PRIOR): a protocol for development of a reporting guideline for overviews of reviews of healthcare interventions. Syst Rev. 2019;8(1):335.
8. Hennessy EA, Johnson BT, Keenan C. Best practice guidelines and essential methodological steps to conduct rigorous and systematic meta-reviews. Appl Psychol Heal Well-Being. 019;11(3):353–81.
9. Pollock M, Fernandes RM, Becker LA, Featherstone R, Hartling L. What guidance is available for researchers conducting overviews of reviews of healthcare interventions? A scoping review and qualitative metasummary. Syst Rev. 2016;5(1):190.
10. Lunny C, Brennan SE, Reid J, McDonald S, McKenzie JE. Overviews of reviews incompletely report methods for handling overlapping, discordant, and problematic data. J Clin Epidemiol . 2020;118:69–85.
11. Gates M, Gates A, Guitard S, Pollock M, Hartling L. Guidance for overviews of reviews continues to accumulate, but important challenges remain: a scoping review. Syst Rev. 2020;9(1):254.
12. Pieper D, Buechter R, Jerinic P, Eikermann M. Overviews of reviews often have limited rigor: a

systematic review. J Clin Epidemiol. 2012;65(12):1267–73.

13. Li L, Tian J, Tian H, Sun R, Liu Y, Yang K. Quality and transparency of overviews of systematic reviews. J Evid Based Med. 2012;5(3):166–73.

14. Gates M, Gates A, Pieper D, Fernandes RM, Tricco, AC, Moher D, et al. Reporting guideline for overviews of reviews of healthcare interventions: development of the PRIOR statement. BMJ. 2022;378, e070849. https://doi.org/10.1136/bmj-2022-070849.

15. Munn Z, Peters MDJ, Stern C, Tufanaru C, McArthur A, Aromataris E. Systematic review or scoping review? Guidance for authors when choosing between a systematic or scoping review approach. BMC Med Res Methodol. 2018;18(1):143.

16. Munn Z. Scoping vs systematic review [video]. YouTube; 2021 [cited 2022 Feb 24]. Available from: https://www.youtube.com/watch?v=BlFExKNmvCM&list=PL1f2FKzk8M6aEifTszswQgEONTsbPkzEm&index=4&t=6s.

17. Tricco AC, Lillie E, Zarin W, O'Brien K, Colquhoun H, Kastner M, et al. A scoping review on the conduct and reporting of scoping reviews. BMC Med Res Methodol. 2016;16:15.

18. Tricco AC, Lillie E, Zarin W, O'Brien KK, Colquhoun H, Levac D, et al. PRISMA extension for scoping reviews (PRISMA-ScR): checklist and explanation. Ann Intern Med. 2018;169(7):467–73.

19. Peters M, Godfrey C, Munn Z, Tricco AC, Khalil H. Chapter 11: Scoping reviews. In Aromataris E, Munn Z, (Eds.). *JBI Manual for Evidence Synthesis [internet]*. JBI; 2020 [cited 2022 Mar 22]. Available from: https://synthesismanual.jbi.global.

20. Peters MDJ, Godfrey CM, Khalil H, McInerney P, Parker D, Soares CB. Guidance for conducting systematic scoping reviews. Int J Evid Based Healthc. 2015;13(3):141–6.

21. Garritty CM, Norris SL, Moher D. Developing WHO rapid advice guidelines in the setting of a public health emergency. J Clinical Epidemiol. 2017;82:47-60.

22. Tricco AC, Langlois EV, Straus SE. Rapid reviews to strengthen health policy and systems: a practical guide [internet]. WHO; 2017 [cited 2022 Nov 24]. Available from: https://apps.who.int/iris/bitstream/handle/10665/258698/9789241512763-eng.pdf.

23. Dobbins M. Steps for conducting a rapid review. Rapid review guidebook [internet]. National Collaborating Centre for Methods and Tools; 2017 [cited 2022 Nov 24]. Available from: https://www.nccmt.ca/uploads/media/media/0001/01/a816af720e4d587e13da6bb307df8c907a5dff9a.pdf.

24. Elliott JH, Synnot A, Turner T, Simmonds M, Akl EA, McDonald S, et al. Living systematic review: 1. Introduction—the why, what, when, and how. J Clin Epidemiol. 2017;91:23–30.

25. Elliott JH, Turner T, Clavisi O, Thomas J, Higgins JPT, Mavergames C, et al. Living systematic reviews: an emerging opportunity to narrow the evidence-practice gap. PLoS Med. 2014;11(2):e1001603.

26. Cochrane Community. Living systematic reviews. Cochrane; 2022 [cited 2022 Mar 24]. Available from: https://community.cochrane.org/review-production/production-resources/living-systematic-reviews.

27. Créquit P, Boutron I, Meerpohl J, Williams HC, Craig J, Ravaud P. Future of evidence

ecosystem series: 2. current opportunities and need for better tools and methods. J Clin Epidemiol. 2020;123:143–52.

28. Riaz IB, He H, Ryu AJ, Siddiqi R, Naqvi SAA, Yao Y, et al. A living, interactive systematic review and network meta-analysis of first-line treatment of metastatic renal cell carcinoma [formula presented]. Eur Urol. 2021;80(6):712–23.

29. Créquit P, Trinquart L, Ravaud P. Live cumulative network meta-analysis: protocol for second-line treatments in advanced non-small-cell lung cancer with wild-type or unknown status for epidermal growth factor receptor. BMJ Open. 2016;6(8):e011841.
